# Gut mycobiota dysbiosis and systemic immune dysfunction in Chinese schizophrenia patients with metabolic syndrome

**DOI:** 10.3389/fimmu.2025.1652633

**Published:** 2025-09-03

**Authors:** Zongxin Ling, Yiwen Cheng, Zhiyong Lan, Xia Liu, Zhangcheng Zhu, Wenwen Ding, Xiaocui Xu, Pian Yu, Xiaoxun Xu, Li Shao, Qinghai Song, Rongxian Liao

**Affiliations:** ^1^ State Key Laboratory for Diagnosis and Treatment of Infectious Diseases, National Clinical Research Center for Infectious Diseases, China-Singapore Belt and Road Joint Laboratory on Infection Research and Drug Development, National Medical Center for Infectious Diseases, Collaborative Innovation Center for Diagnosis and Treatment of Infectious Diseases, The First Affiliated Hospital, Zhejiang University School of Medicine, Hangzhou, Zhejiang, China; ^2^ Yuhang Institute for Collaborative Innovation and Translational Research in Life Sciences and Technology, Hangzhou, Zhejiang, China; ^3^ Jinan Microecological Biomedicine Shandong Laboratory, Jinan, Shandong, China; ^4^ Department of Psychiatry, Quzhou Third Hospital, Quzhou, Zhejiang, China; ^5^ Department of Intensive Care Unit, The First Affiliated Hospital, Zhejiang University School of Medicine, Hangzhou, Zhejiang, China; ^6^ Department of Preventive Medicine, School of Public Health and Management, Wenzhou Medical University, Wenzhou, Zhejiang, China; ^7^ Department of Anesthesiology, Affiliated Hospital of Nantong University, Medical School of Nantong University, Nantong, Jiangsu, China; ^8^ School of Clinical Medicine, The Affiliated Hospital of Hangzhou Normal University, Hangzhou, Zhejiang, China; ^9^ Department of Psychiatry, Lishui Second People’s Hospital, Lishui, Zhejiang, China

**Keywords:** schizophrenia, metabolic syndrome, mycobiota, gut-brain axis, immunological dysfunction

## Abstract

While bacterial dysbiosis has been extensively studied in schizophrenia with metabolic syndrome (SZ-MetS), the role of gut mycobiota in this comorbidity remains unclear. This study represents the first comprehensive investigation of fungal communities in SZ-MetS patients (n=109) versus healthy controls (HCs, n=101) using ITS1 sequencing and multi-parameter immune profiling. Although global mycobiota structure showed no significant differences, compositional analyses revealed profound taxonomic shifts: pathobionts (*Trichosporon asahii*, *Candida albicans*, *Lodderomyces elongisporus*) were enriched, while putative beneficial species (*Saccharomyces cerevisiae*, *Pleurotus ostreatus*) were reduced in patients. Enterotyping identified two mycobiota clusters (*Candida*-dominant vs *Aspergillus*-dominant), though their distribution was similar between groups. Notably, machine learning revealed a six-species fungal signature with strong diagnostic potential (AUC = 0.86). Species-specific immune correlations were also observed: inflammatory cytokines such as IL-6 and MIP-1α were positively associated with *Ustilago esculenta* and *Trichosporon asahii*, but negatively correlated with *Saccharomyces cerevisiae*. Furthermore, fungal abundances were differentially correlated with metabolic and psychiatric parameters, with *Lodderomyces* linked to elevated triglycerides and *S. cerevisiae* associated with reduced symptom severity. These findings reveal that while overall fungal community structure is preserved, SZ-MetS exhibits distinct mycobiota alterations that interact with host immunity and clinical manifestations, suggesting fungi may contribute to the SZ-MetS vicious cycle through taxon-specific mechanisms.

## Introduction

Schizophrenia (SZ) is a severe neuropsychiatric disorder characterized by cognitive impairment and a reduced life expectancy of 15–20 years, attributable in part to its frequent comorbidity with metabolic syndrome (MetS) (1, 2). Approximately one-third of SZ patients develop MetS (3), creating a vicious cycle that exacerbates psychiatric symptoms, accelerates cognitive decline, and increases cardiovascular mortality (4–6). While antipsychotic side effects and lifestyle factors contribute to this comorbidity (7), emerging evidence implicates gut microbiome dysbiosis as a potential unifying mechanism bridging metabolic and neuropsychiatric pathology. Extensive research has characterized bacterial dysbiosis in both SZ and MetS separately, revealing altered microbial diversity, short-chain fatty acid production, and gut barrier integrity (8–14). Our previous work demonstrated distinct fecal bacterial profiles in SZ-MetS patients correlating with immune dysfunction (12). However, the fungal microbiome (mycobiota) - an equally important component of the gut ecosystem - remains virtually unexplored in SZ-MetS comorbidity, despite its known roles in immune modulation and metabolic regulation (15–17).

Fungal-bacterial interactions critically maintain gut homeostasis, with mycobiota dysbiosis implicated in inflammatory diseases and metabolic disorders (18–20). Notably, *Candida albicans* overgrowth associates with obesity and insulin resistance (21), while *Saccharomyces* species modulate host immunity in ways relevant to neuroinflammation (22). The unique ability of fungi to form biofilms and penetrate intestinal barriers may make them particularly potent modulators of the gut-brain axis (23). Nevertheless, no studies to date have investigated gut mycobiota alterations in the SZ-MetS overlap, representing a critical knowledge gap given the established links between fungal dysbiosis, systemic inflammation, and neuropsychiatric conditions.

This study represents the first comprehensive analysis of gut fungal communities in Chinese SZ patients with MetS. We enrolled hospitalized SZ-MetS patients and age- and gender-matched healthy controls from Quzhou, China. Using internal transcribed spacer 1 (ITS1) sequencing and multi-parameter immunoassays, we characterize mycobiota profiles distinguishing SZ-MetS patients from matched controls; and examine correlations between fungal taxa and peripheral immune markers. Our findings may reveal novel mycobiota signatures contributing to the SZ-MetS vicious cycle, potentially informing future microbiome-targeted interventions for this high-risk population.

## Materials and methods

### Participants’ enrollment and sample collection

Building upon our established cohort (12), this study ultimately included 109 SZ-MetS patients (age 28–64 years) and 101 age- and gender-matched healthy controls (HCs) recruited from Quzhou Third People’s Hospital between June and November 2023. The study protocol received ethical approval from the hospital’s Institutional Review Board (approval no. SY-2023-17), and all participants or their legal guardians provided written informed consent prior to enrollment.

We adhered to the same inclusion and exclusion criteria, as well as participant characteristics, as detailed in our previous work (12). Notably, individuals with recent use (within 1 month) of any antimicrobial medications—including antibacterial, antifungal, antiparasitic, or antiviral agents—were excluded. For mycobiota analysis, participants provided approximately 2g of fresh fecal samples collected in sterile containers, which were immediately flash-frozen at -80°C within 15 minutes of collection to ensure microbial stability. Concurrently, we obtained fasting venous blood samples during morning hours, with plasma separation completed within 15 minutes of collection followed by storage at -80°C until analysis.

### ITS1 sequencing and bioinformatic analysis

Fungal DNA was isolated from 300 mg aliquots of homogenized fecal samples using the QIAamp^®^ DNA Stool Mini Kit (QIAGEN, Germany), with mechanical lysis enhancement through glass-bead disruption (Mini-beadbeater; Thermo Electron Corporation, USA). We amplified the ITS1 region using fungal-specific primers (ITS1F: 5’-CTTGGTCATTTAGAGGAAGTAA-3’; ITS2R: 5’-GCTGCGTTCTTCATCGATGC-3’) to construct sequencing libraries. All library preparation and subsequent paired-end sequencing on the Illumina NovaSeq 6000 platform were conducted by Hangzhou KaiTai Bio-lab’s technical team, following established protocols (20).

We processed sequencing data using QIIME2 (v2020.11) with the following workflow: First, raw sequences underwent adapter and barcode removal using Cutadapt (v2.4), followed by quality filtering and chimera elimination through DADA2 to generate high-quality amplicon sequence variants (ASVs) (24). Taxonomic classification was performed against the UNITE database (Release 9.0, http://unite.ut.ee/index.php) after normalizing samples to equal sequencing depth. For community analysis, we calculated α-diversity (within-sample richness) and β-diversity (between-sample differences) metrics following established fungal microbiota protocols (13, 19, 25). Fungal enterotype clustering revealed distinct community structures across samples. To identify diagnostic fungal signatures, we implemented a Random Forest classifier (Mean Decrease Gini for feature importance) and validated discriminative performance using ROC curve analysis via OECloud platform (https://cloud.oebiotech.com).

### Systemic immune function analysis

To evaluate participants’ systemic immune function, we employed a 27-plex human group I cytokine assay kit (Bio-Rad, CA, USA) following our previously established methodology (12). This magnetic bead-based immunoassay quantified 27 cytokines and chemokines—including 16 cytokines, 6 chemokines, and 5 growth factors—according to the manufacturer’s protocols. Analysis was performed using the Bio-Plex 200 system, with data processed via Bio-Plex Manager v5.0 software. Results were expressed as picograms per milliliter (pg/mL) using integrated standard curves, yielding intra- and inter-assay coefficient of variation (CV) values of 5–8% for reproducibility.

### Statistical analysis

For statistical analyses, continuous variables (e.g., α-diversity indices, microbial taxonomic abundances, cytokine levels) were evaluated using independent *t*-tests or Mann-Whitney *U* tests based on normality assessments (Shapiro-Wilk test). Categorical variables were compared via Pearson’s chi-square test or Fisher’s exact test. Spearman’s rank correlation was used to assess associations between microbial abundances, cytokine profiles, and clinical parameters. Differential abundance analyses of microbial taxa were performed using STAMP v2.1.3 with Welch’s *t*-test or nonparametric tests, while α-diversity indices (e.g., Chao1, Shannon) were compared using permutation tests. All statistical analyses were conducted in SPSS v24.0 and R packages (e.g., phyloseq, vegan), with visualizations created using GraphPad Prism v6.0. Multiple comparisons were adjusted via the Benjamini-Hochberg procedure to control false discovery rate (FDR), with statistical significance set at FDR < 0.05.

## Results

### Gut mycobiota characteristics in SZ-MetS patients

**Figure 1:**
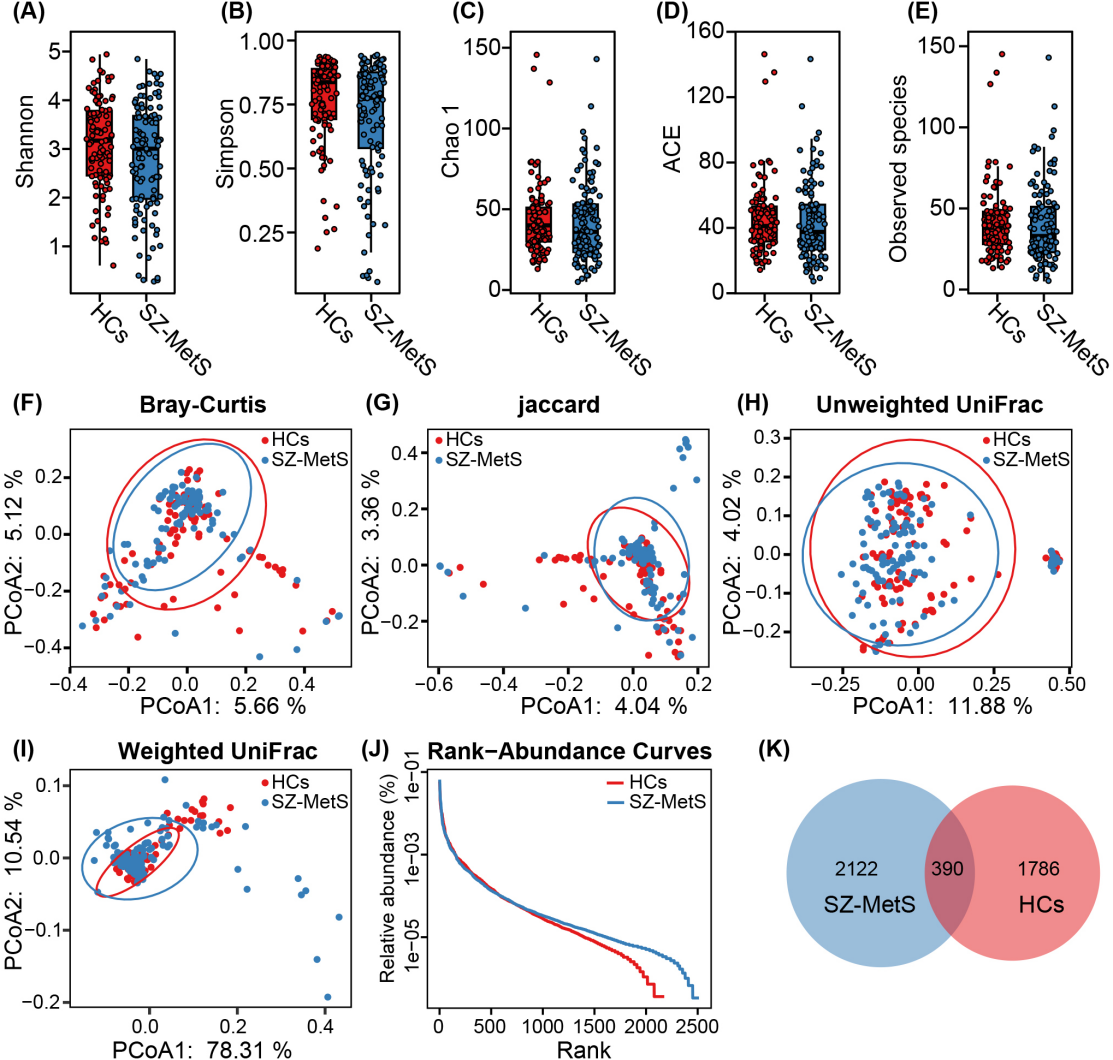
Comparison of fecal mycobiota structure between SZ-MetS patients and healthy controls. **(A–E)** α-diversity indices (Shannon and Simpson) and richness indices (Chao1, ACE, and observed species) were used to assess the overall structure of the fecal mycobiota, with data presented as mean ± standard deviation. Unpaired two-tailed t-tests were performed for inter-group comparisons. **(F–I)** Principal coordinate analysis (PCoA) plots illustrating β-diversity of individual fecal mycobiota based on Bray–Curtis, Jaccard, unweighted UniFrac, and weighted UniFrac distances, with each symbol representing an individual sample. **(J)** The rank-abundance curve of fungal amplicon sequence variants (ASVs) shows a higher presence of low-abundance ASVs in the fecal mycobiota of SZ-MetS patients compared to healthy controls. **(K)** Venn diagram illustrating the overlap of ASVs in the fecal mycobiota of SZ-MetS patients and healthy controls.

We performed comprehensive characterization of gut fungal communities in our cohort of 109 SZ-MetS patients and 101 matched HCs. Following quality filtering of 24,380,498 raw ITS1 sequences, we retained 20,739,649 high-quality reads with an average of 102,532 reads per sample for downstream analysis. After normalizing to an even sequencing depth of 27,214 reads per sample, we identified 4,298 ASVs, with SZ-MetS patients exhibiting 15.5% more ASVs than controls (2,512 vs 2,176). Fecal mycobiota diversity was compared between SZ-MetS patients and HCs based on the relative ASVs table (**Figure 1**). Our analysis revealed no significant structural differences in gut fungal communities between SZ-MetS patients and HCs. Alpha diversity metrics showed comparable fungal diversity between groups, with no significant differences observed in diversity indices (Shannon and Simpson; p > 0.05; **Figures 1A, B**) and richness estimators (ACE, Chao1, and Observed species; p > 0.05; **Figures 1C–E**). Similarly, beta diversity analyses using multiple distance metrics such as Bray-Curtis, Jaccard, unweighted UniFrac, and weighted UniFrac algorithms indicated no significant overall differences in community composition between SZ-MetS patients and HCs (all ADONIS p>0.05, **Figures 1F–I**). Notwithstanding these similarities, we identified important compositional distinctions. Rank-abundance curves revealed comparable species distributions (**Figure 1J**), while Venn analysis demonstrated a significant enrichment of unique ASVs in SZ-MetS patients (2122 vs 1786 in HCs) (**Figure 1K**). These findings suggest that while global mycobiota diversity and structure remain similar, SZ-MetS patients exhibit an expanded repertoire of unique fungal species that may contribute to disease pathophysiology.

### Gut mycobiota composition and enterotype analysis in SZ-MetS patients

**Figure 2:**
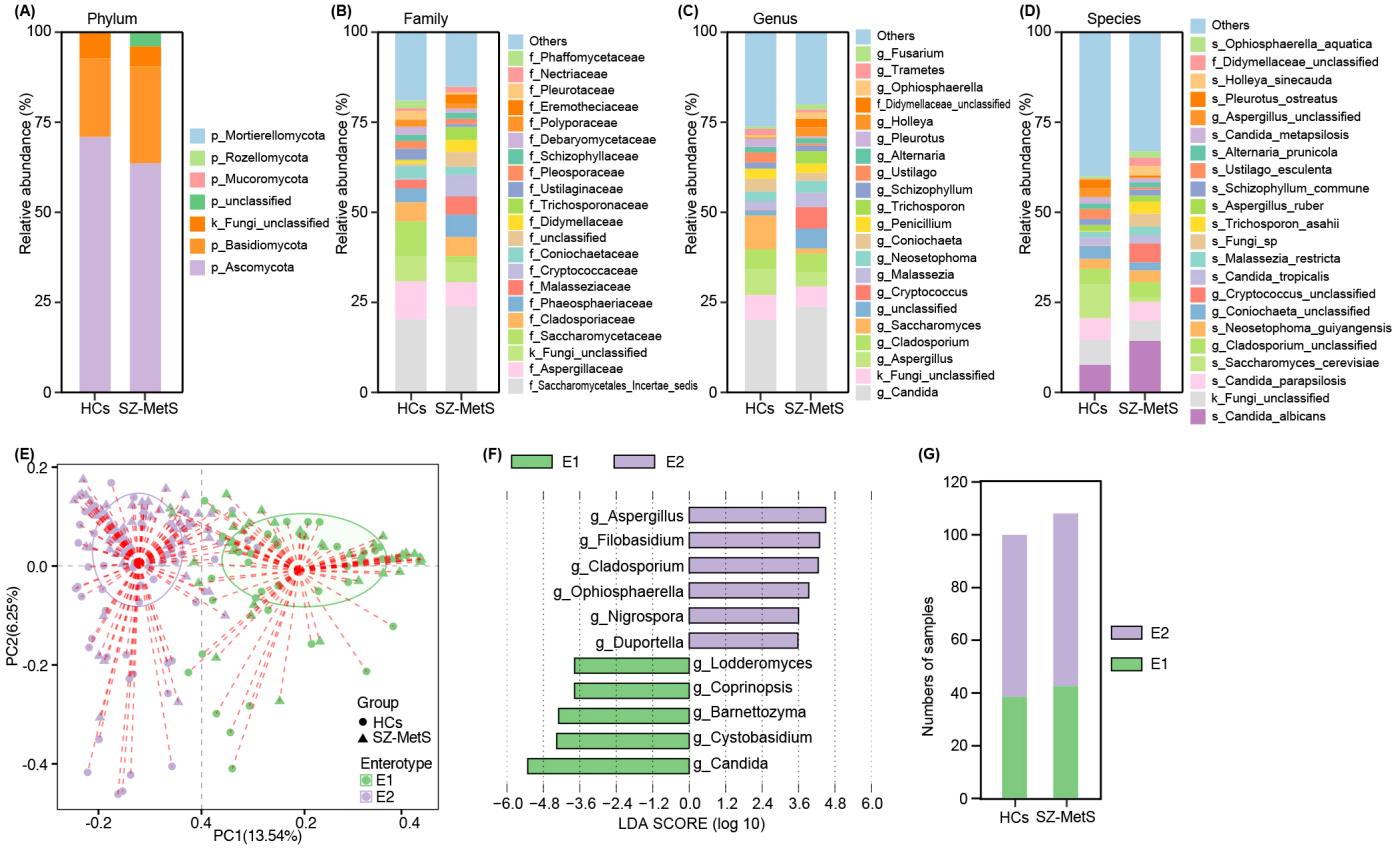
Gut mycobiota composition in SZ-MetS patients and healthy controls. **(A)** Phylum; **(B)** Family; **(C)** Genus; **(D)** Species; **(E)** PCoA plot identifying two enterotypes; **(F)** LEfSe analysis of enterotype-specific genera; **(G)** Enterotype distribution.

Using the UNITE database, ITS sequencing reads were taxonomically assigned to 5 phyla, 158 families, 292 genera, and 478 species. Taxonomic profiling revealed significant differences in gut mycobiota composition between SZ-MetS patients and HCs across various taxonomic levels. At the phylum level, both groups were primarily dominated by Ascomycota and Basidiomycota (**Figure 2A**). Family-level analysis identified differentially abundant taxa with clinical relevance: Saccharomycetales_Incertae_sedis and Phaeosphaeriaceae were enriched in SZ-MetS patients, whereas Aspergillaceae and Pleosporaceae showed decreased abundance compared to HCs (**Figure 2B**). Genus-level profiling further highlighted significant shifts in *Candida*, *Aspergillus*, *Saccharomyces*, and *Cladosporium* (**Figure 2C**), with species-specific analyses confirming these trends (**Figure 2D**). Enterotype analysis classified the cohort into two distinct mycobiota clusters: Enterotype 1 (E1), characterized by *Candida* dominance, and Enterotype 2 (E2), marked by *Aspergillus* prevalence (**Figures 2E, F**). However, the distribution of these enterotypes did not differ significantly between groups (HC: 39 E1/62 E2 vs SZ-MetS: 43 E1/66 E2; **Figure 2G**). These findings demonstrate that while SZ-MetS patients exhibit distinct shifts in specific fungal taxa, the overall enterotype distribution remains comparable to HCs, suggesting that disease-associated mycobiota alterations may occur independently of broader community stratification.

### Comparative analysis of gut mycobiota between SZ-MetS patients and HCs

**Figure 3:**
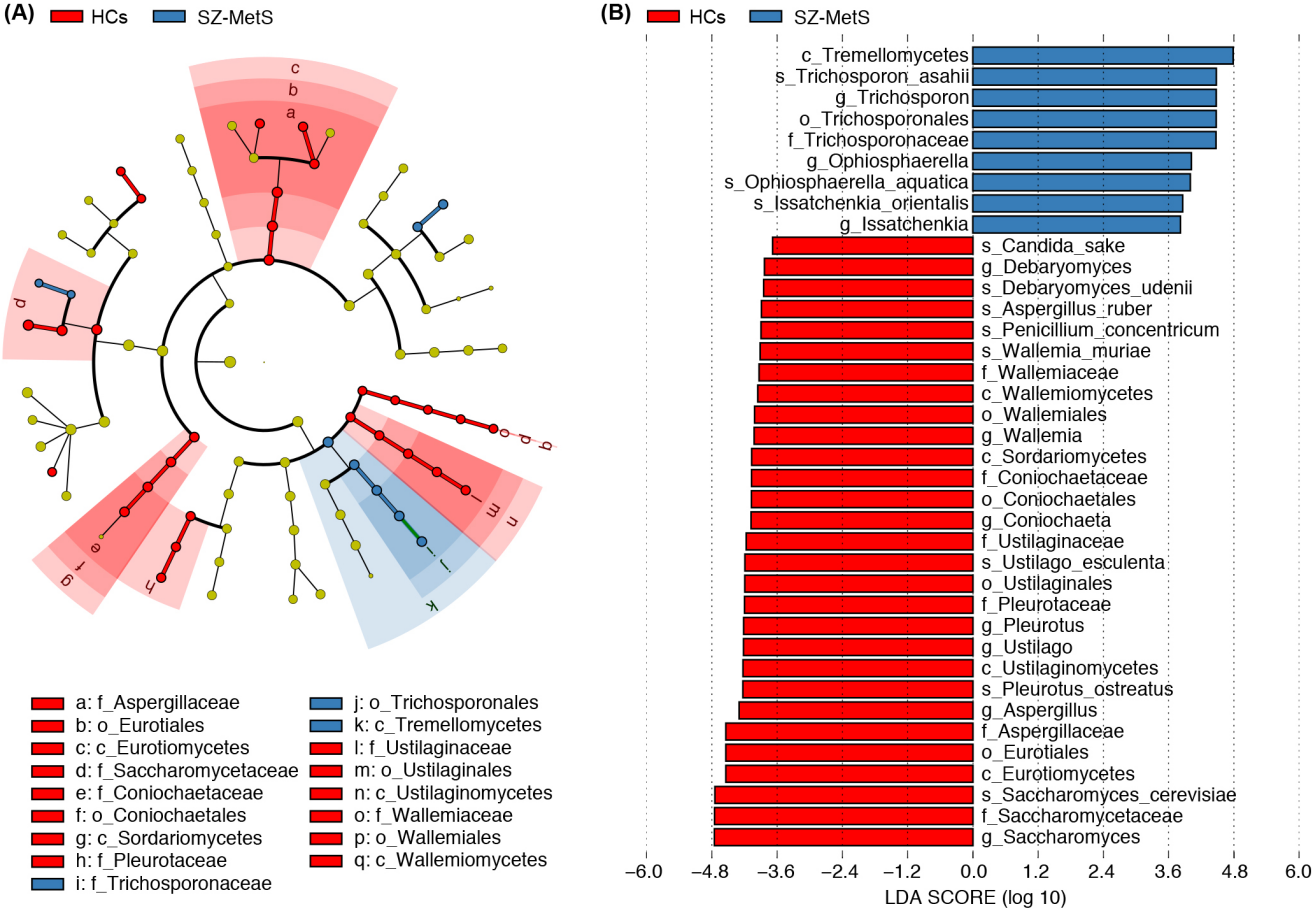
Differential fecal mycobiota between SZ-MetS patients and healthy controls. **(A)** LEfSe cladograms showing fungal taxa significantly associated with SZ-MetS patients or healthy controls. Circle size indicates relative abundance, with circles representing taxonomic levels from phylum to species. Statistical significance was determined by Wilcoxon rank-sum test (p < 0.05). **(B)** Histogram of Linear Discriminant Analysis (LDA) scores (> 3.5) for fungal taxa with the largest abundance differences between SZ patients and healthy controls (p < 0.05).

LEfSe analysis revealed significant compositional differences in gut fungal communities between SZ-MetS patients and HCs. The cladogram visually highlights differentially abundant taxa across all taxonomic levels, from phylum to species (**Figure 3A**). Notably, several fungal species showed significant enrichment in SZ-MetS patients, including *Issatchenkia orientalis*, *Ophiosphaerella aquatica*, and *Trichosporon asahii*. Conversely, multiple potentially beneficial species were significantly reduced in patients, including *Saccharomyces cerevisiae*, *Pleurotus ostreatus*, *Ustilago esculenta*, *Wallemia muriae*, *Penicillium concentricum*, *Aspergillus ruber*, *Debaryomyces udenii*, and *Candida sake* (**Figure 3B**).

**Figure 4:**
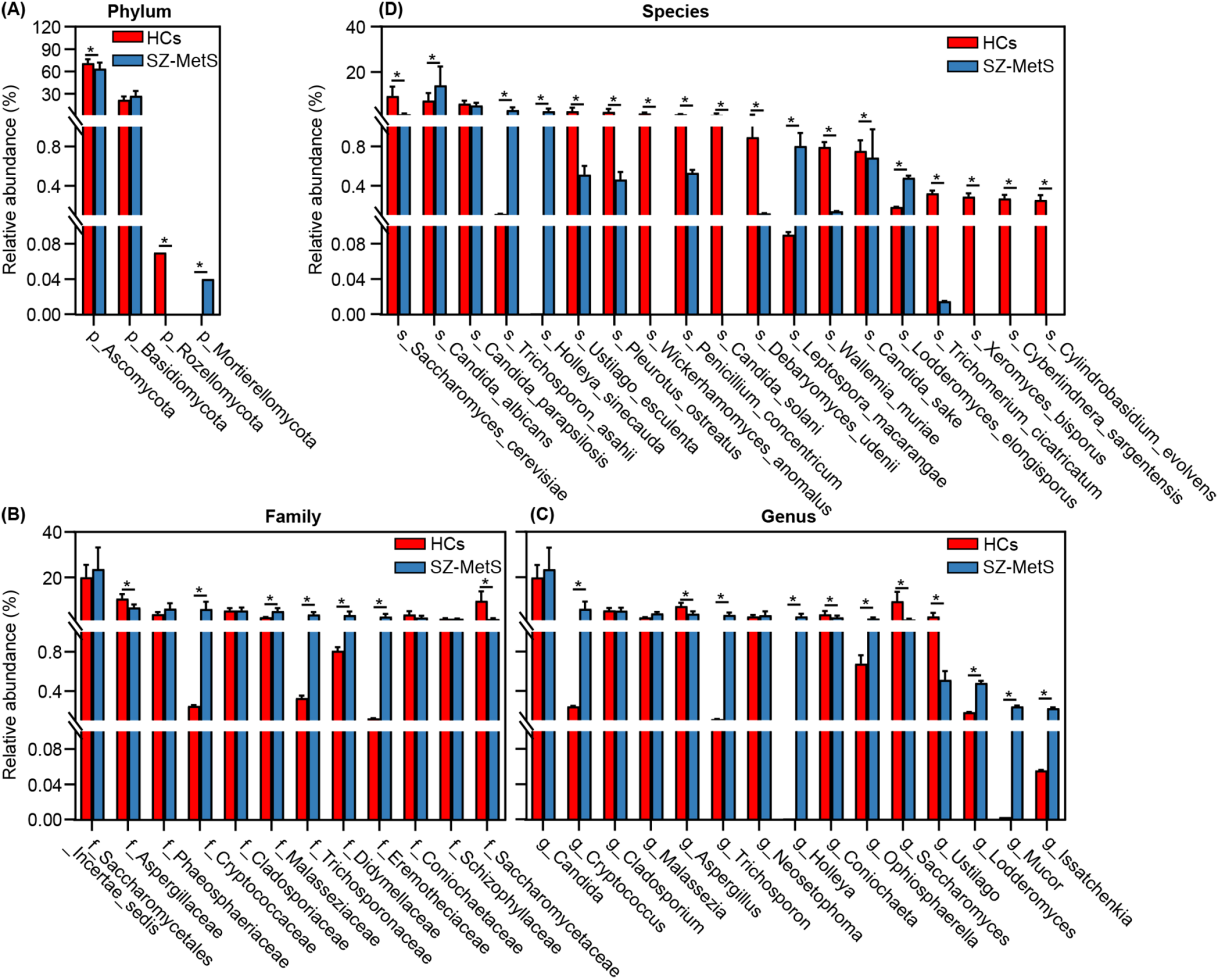
MetaStats2 confirmed key differential fecal fungal taxa between SZ-MetS patients and healthy controls. **(A)** Differential functional phyla; **(B)** Differential functional families; **(C)** Differential functional genera; **(D)** Differential functional species. Data are presented as mean ± standard deviation. Mann–Whitney *U*-tests were used for comparisons between SZ-MetS patients and healthy controls. *p < 0.05 vs. control group.

**Figure 5:**
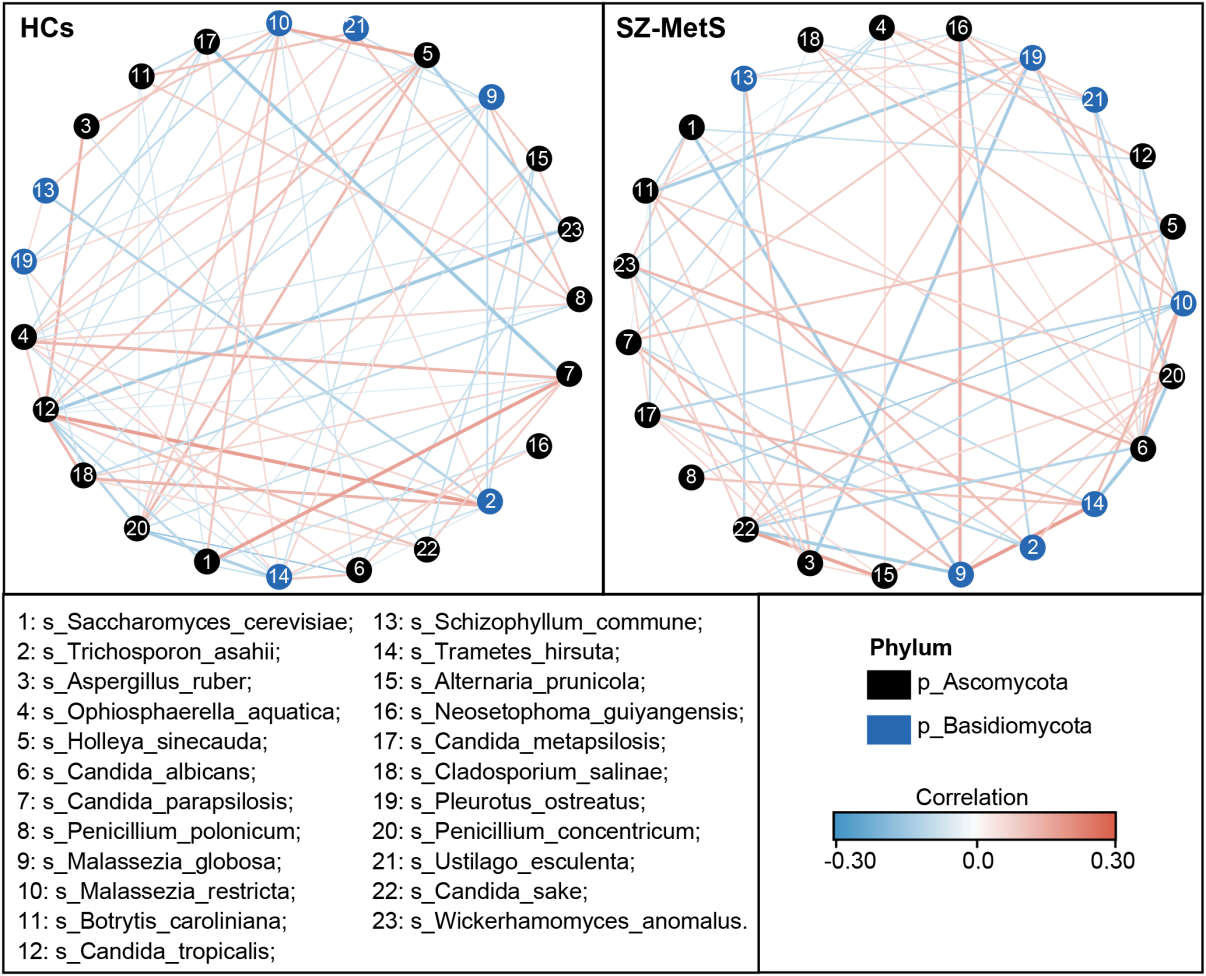
Co-occurrence network of abundant fecal genera in SZ-MetS patients and controls. The co-occurrence network was constructed using the SparCC algorithm on relative abundance data at the genus level, illustrating ecological interactions within the microbial community. Cytoscape version 3.6.1 was used for network construction. Red and blue lines represent positive and negative correlations, respectively.

Subsequently, we compared the fecal mycobiota between the two groups at specific taxonomic levels using MetaStats 2.0. At the phylum level, SZ-MetS patients exhibited a decrease in Ascomycota and Rozellomycota, while showing an increased abundance of Mortierellomycota (**Figure 4A**). At the family level, five families—Cryptococcaceae, Malasseziaceae, Trichosporonaceae, Didymellaceae, and Eremotheciaceae—were elevated in SZ-MetS patients, while two families, Aspergillaceae and Saccharomycetaceae, were significantly reduced (**Figure 4B**). At the genus level, seven genera, including *Cryptococcus*, *Trichosporon*, *Holleya*, *Ophiosphaerella*, *Lodderomyces*, *Mucor*, and *Issatchenkia*, were more abundant in SZ-MetS patients, while four genera—*Aspergillus*, *Coniochaeta*, *Saccharomyces*, and *Ustilago*—were reduced (**Figure 4C**). At the species level, *Candida albicans*, *Trichosporon asahii*, *Holleya sinecauda*, *Leptospora macarangae*, and *Lodderomyces elongisporus* were more abundant in SZ-MetS patients, whereas *Saccharomyces cerevisiae*, *Ustilago esculenta*, *Pleurotus ostreatus*, *Wickerhamomyces anomalus*, *Penicillium concentricum*, and *Candida solani* were significantly reduced (**Figure 4D**). Additionally, network analysis using SparCC revealed notable differences in fungal ecological relationships, with SZ-MetS patients exhibiting a simpler co-occurrence network compared to HCs (**Figure 5**). This reduced complexity was especially pronounced among Ascomycota species, indicating disrupted fungal community stability.

**Figure 6:**
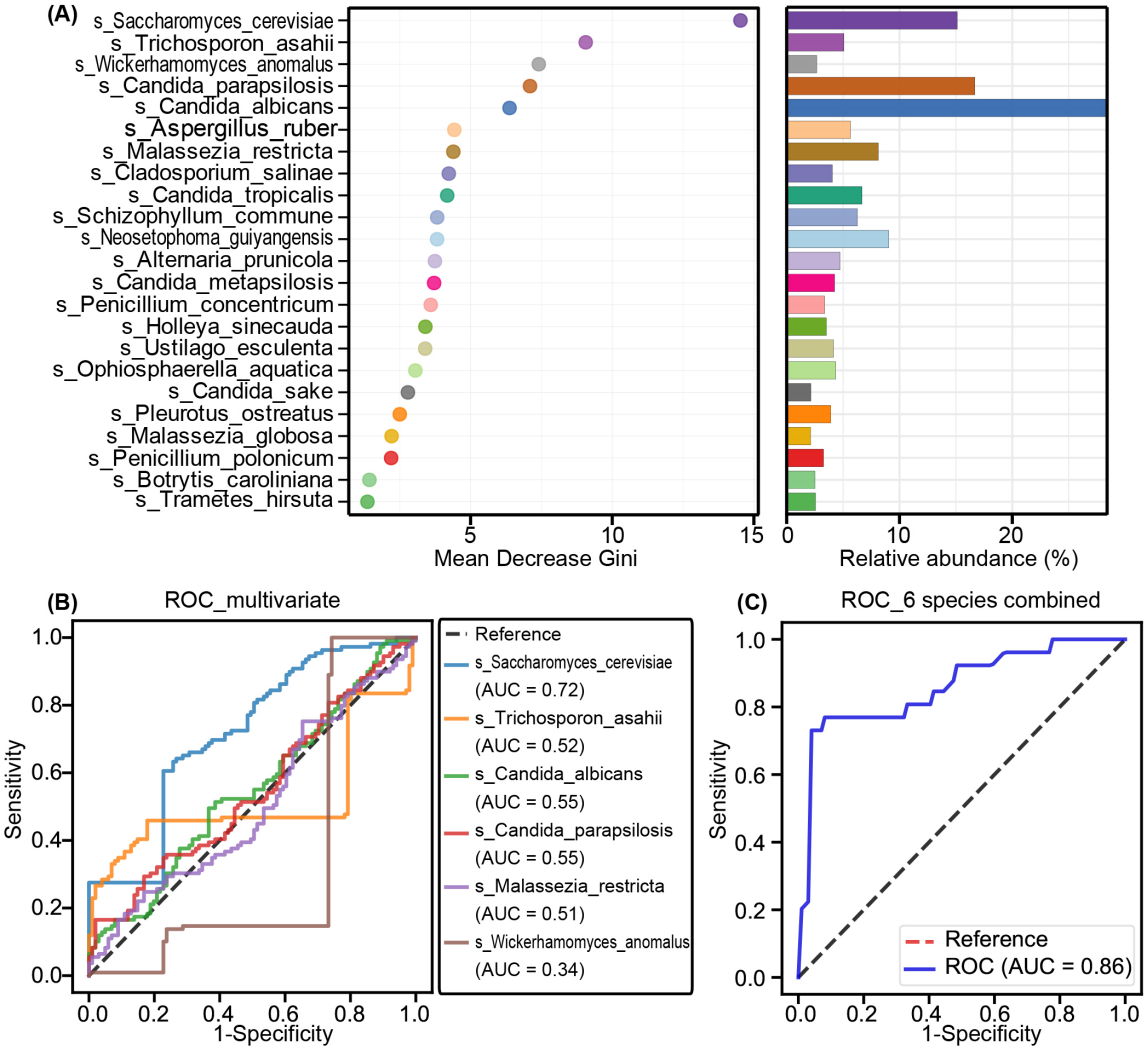
Fungal biomarkers for SZ-MetS diagnosis. **(A)** Random Forest analysis highlighting the importance of various fungal species based on Mean Decrease Gini. **(B)** Receiver Operating Characteristic (ROC) curves for individual fungal species to distinguish SZ-MetS patients from healthy controls. **(C)** ROC curves for a combination of six fungal species to distinguish SZ-MetS patients from healthy controls. AUC represents the area under the ROC curve.

We also assessed the potential of key functional fungal species to distinguish SZ-MetS patients from HCs using Random Forest and ROC analysis. Random Forest, a machine learning classification model, helps classify microbial community samples by evaluating the importance of variables using Mean Decrease Gini. A higher value indicates greater significance of a species (**Figure 6A**). ROC curves were then generated to evaluate the diagnostic performance of fungal species at the species level, with AUC values reflecting their accuracy. Among the differential species, *Saccharomyces cerevisiae* showed moderate diagnostic value (AUC = 0.72), while *Trichosporon asahii*, *Candida albicans*, and *Candida parapsilosis* had limited discriminatory power (AUC = 0.52–0.56) (**Figure 6B**). However, the combined model of six species significantly improved diagnostic accuracy (AUC = 0.86), demonstrating the potential of multivariate approaches in fungal biomarker analysis (**Figure 6C**). These findings reveal a profound dysbiosis in the gut mycobiota of SZ-MetS patients, characterized by both the expansion of specific fungal taxa and depletion of potentially protective commensals, suggesting possible implications for disease pathophysiology and diagnostic development.

### Association between key fungal species and systemic inflammatory/clinical markers

**Figure 7:**
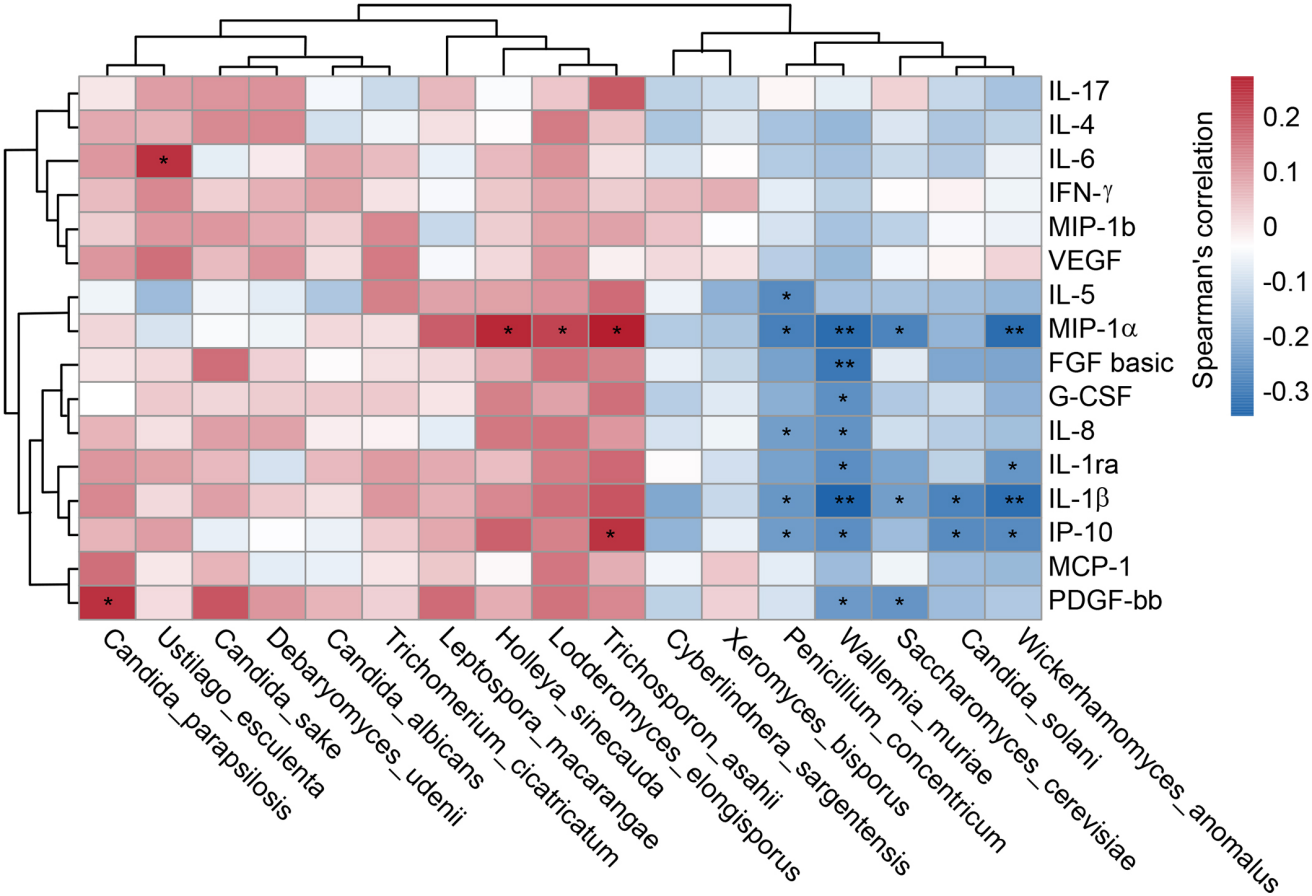
Gut fungal species linked to systemic immune dysfunction in SZ-MetS patients. Spearman’s correlation heatmap depicting associations between differentially abundant fecal fungal species and circulating immune markers (inflammatory cytokines, chemokines, and growth factors) in SZ-MetS patients. Color intensity represents the strength of correlation, with significance thresholds indicated (p < 0.05). Only statistically significant correlations are annotated. *p < 0.05; **p < 0.05.

**Figure 8:**
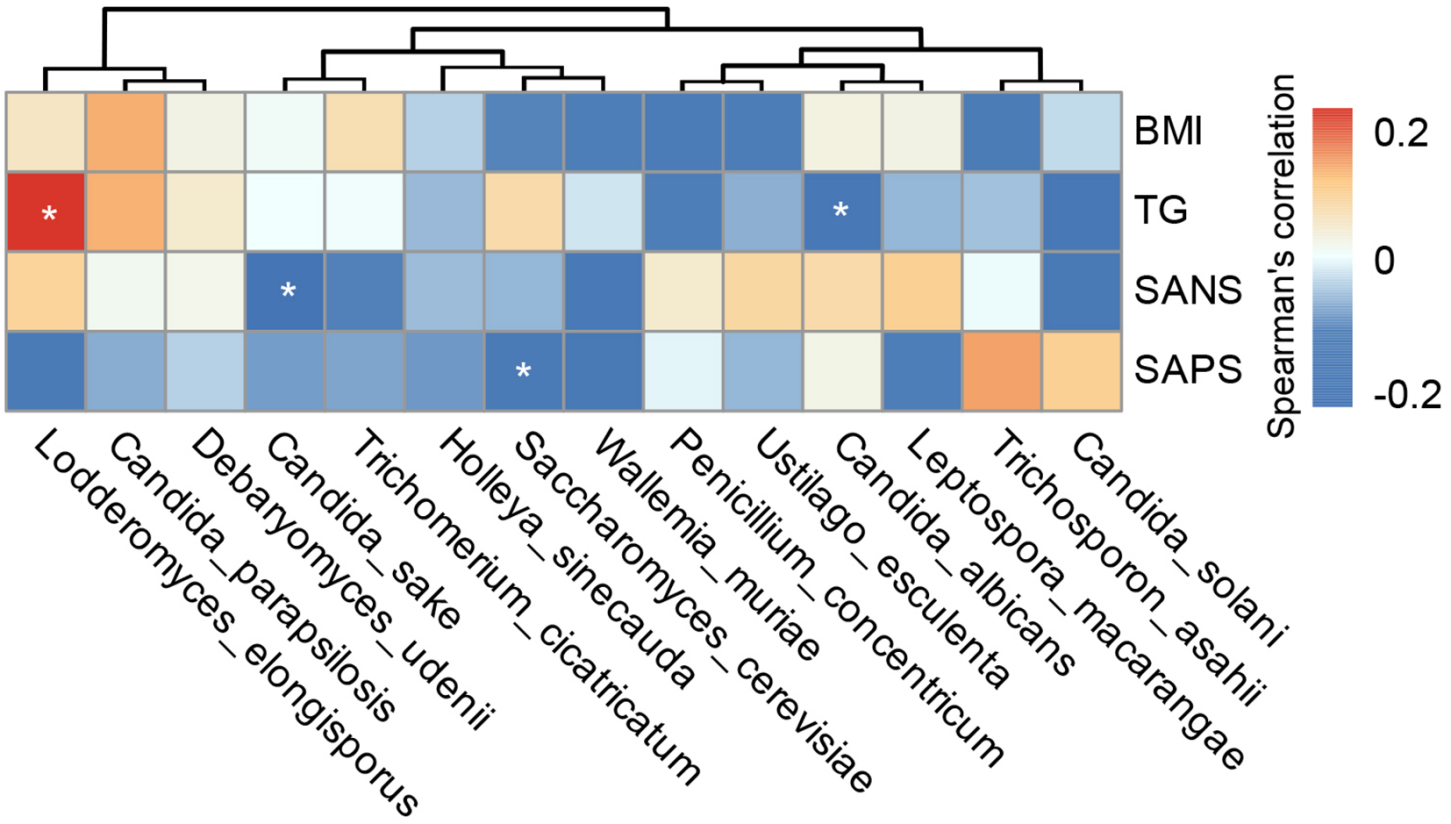
Associations between gut fungal species and clinical indicators in SZ-MetS patients. Heatmap of Spearman’s rank correlations between differentially abundant gut fungal species and clinical parameters in SZ-MetS patients. Significant positive correlations (p < 0.05) are shown in red, significant negative correlations (p < 0.05) in blue, and non-significant correlations (p ≥ 0.05) in white. *p < 0.05.

Our multiplex cytokine analysis revealed a complex immune dysfunction in SZ-MetS, characterized by elevated levels of several inflammatory cytokines and chemokines relative to HCs. Specifically, the concentrations of cytokines in SZ-MetS versus HCs were as follows: such as IL-1β (3.14 ± 3.03 pg/mL vs. 0.94 ± 0.31 pg/mL), IL-1ra (650.79 ± 546.49 pg/mL vs. 246.95 ± 125.22 pg/mL), IL-4 (3.84 ± 2.08 pg/mL vs. 2.59 ± 0.8 pg/mL), IL-5 (68.69 ± 45.93 pg/mL vs. 27.1 ± 25.94 pg/mL), IL-6 (12.07 ± 10.07 pg/mL vs. 4.18 ± 2.07 pg/mL), IL-8 (111.94 ± 162.57 pg/mL vs. 10.26 ± 6.77 pg/mL), IL-17 (35.36 ± 14.78 pg/mL vs. 28.03 ± 6.69 pg/mL), and IFN-γ (8.89 ± 4.13 pg/mL vs. 6.25 ± 1.36 pg/mL). Similarly, chemokine levels in SZ-MetS compared to HCs were: IP-10 (484.46 ± 158.12 pg/mL vs. 316.43 ± 93.4 pg/mL), MCP-1 (30.4 ± 17.81 pg/mL vs. 19.85 ± 10.71 pg/mL), MIP-1α (35.63 ± 22.48 pg/mL vs. 2.66 ± 1.55 pg/mL), and MIP-1β (227.26 ± 106.2 pg/mL vs. 158.34 ± 34.74 pg/mL). Spearman correlation analysis identified significant associations between specific gut fungal species and systemic inflammatory markers as well as clinical parameters. Pro-inflammatory cytokines like IL-6 were positively correlated with *Ustilago esculenta*, while chemokines such as MIP-1α showed positive correlations with *Holleya sinecauda*, *Lodderomyces elongisporus*, and *Trichosporon asahii*, and IP-10 with *Trichosporon asahii*. In contrast, *Saccharomyces cerevisiae*, *Penicillium concentricum*, and *Wickerhamomyces anomalus* exhibited negative correlations with inflammatory markers like MIP-1α and IL-1β, and *Wallemia muriae* negatively correlated with MIP-1, IL-8, IL-1β, and IP-10. Both *Wallemia muriae* and *Wickerhamomyces anomalus* also showed negative correlations with IL-1ra (**Figure 7**). Additionally, *Lodderomyces elongisporus* was positively correlated with metabolic parameters like triglycerides (TG), whereas *Candida albicans* was negatively correlated with TG. Notably, *Saccharomyces cerevisiae* exhibited inverse correlations with Scale for Assessment of Positive Symptoms (SAPS), while *Candida sake* was negatively correlated with Scale for Assessment of Negative Symptoms (SANS) (**Figure 8**). These findings suggest that alterations in gut mycobiota may influence systemic inflammation and clinical manifestations in SZ-MetS patients through species-specific interactions.

## Discussion

The intricate bidirectional relationship between SZ and MetS presents significant clinical challenges, where antipsychotic medications-while crucial for managing psychiatric symptoms - paradoxically contribute to metabolic dysregulation through mechanisms that go beyond their direct pharmacological effects (26, 27). Mounting evidence highlights the gut microbiota as a key mediator in this metabolic-neuropsychiatric crosstalk. Cutting-edge multi-omics research demonstrates that gut microbial imbalances often precede and may drive metabolic abnormalities in SZ patients, regardless of antipsychotic use (12, 28). Often referred to as the “second brain” and “metabolic organ”, the gut microbiota serves as a vital link between nutritional metabolism and neurobehavioral regulation (29). Comprising both bacterial and fungal components, it regulates host metabolism through multiple pathways including short-chain fatty acids (SCFAs) production, maintenance of gut barrier function, and immune system regulation (9, 30, 31). Current understanding has been predominantly shaped by bacterial research, overlooking the potentially crucial contributions of fungal communities (12, 32). The gut mycobiota represents a distinct ecological niche that interacts with host physiology through mechanisms fundamentally different from those of bacteria. Unlike bacterial signaling which primarily occurs through SCFAs production, fungal communities influence host metabolism and immunity through unique pathways via β-glucan recognition by Dectin-1 receptors that modulates both innate and adaptive immune responses (33), production of neuroactive tryptophan metabolites that can cross the blood-brain barrier (34), and direct vagus nerve stimulation capable of altering central nervous system activity (35, 36). These fungal-specific mechanisms may be particularly relevant to SZ-MetS pathogenesis given the established roles of immune activation and neurotransmitter dysregulation in both SZ and MetS. Furthermore, the ability of certain fungal species to form biofilms and penetrate intestinal barriers positions them as potential amplifiers of gut-derived inflammation - a hypothesized contributor to both neuropsychiatric symptoms and metabolic dysfunction. Despite these plausible mechanistic links, the gut mycobiota remains conspicuously understudied in the context of SZ-MetS comorbidity, representing a critical gap in our understanding of how microbial communities influence the gut-brain-metabolism axis. This knowledge deficit persists even as research continues to elucidate bacterial dysbiosis patterns in SZ-MetS, including the depletion of anti-inflammatory butyrate producers and expansion of pro-inflammatory taxa (13, 28, 37, 38). The current study addresses this gap by providing the first systematic investigation of gut mycobiota alterations in SZ-MetS, offering new insights into how fungal communities may contribute to this complex comorbidity through their unique biological properties and host interaction mechanisms.

Our study pioneers the exploration of gut mycobiota in SZ-MetS patients, revealing profound taxonomic alterations despite comparable global α- and β-diversity. Taxonomic profiling uncovered striking shifts in specific fungal taxa, with SZ-MetS patients exhibiting enrichment of *Candida*, *Trichosporon*, and *Issatchenkia* alongside reduction of protective species like *S. cerevisiae* and *P. ostreatus*—findings that align with emerging literature on fungal dysbiosis in metabolic and neuroinflammatory disorders. For instance, *C. albicans* overgrowth, a prevalent species within the *Candida* genus, has been mechanistically linked to insulin resistance in MetS by disrupting gut mucosal barriers, increasing permeability, and promoting endotoxemia that impairs insulin signaling in peripheral tissues (39, 40). Concurrently, the depletion of *S. cerevisiae*—a known modulator of tight-junction proteins—may exacerbate gut leakage (41), while its reduction also disrupts SCFAs production critical for metabolic homeostasis (42, 43). Preclinical models demonstrate that *S. cerevisiae*-depleted mycobiomes correlate with reduced SCFAs levels, dyslipidemia, and altered energy metabolism, mirroring metabolic derangements in SZ-MetS (44). Mechanistically, these fungal alterations may influence neuropsychiatric function through dual pathways: neurotransmitter regulation and immunomodulation. Altered fungi such as *Trichosporon* promote the release of pro-inflammatory cytokines that disrupt neural plasticity, while *S. cerevisiae* depletion may reduce production of neuroactive metabolites like serotonin and GABA (45–47). In animal models, *Saccharomyces boulardii* supplementation restores brain serotonin levels and alleviates neuropsychiatric-like behaviors, underscoring fungi’s direct impact on the gut-brain axis (48). Additionally, fungal dysbiosis triggers abnormal immune activation in SZ-MetS, with *Trichosporon*-induced pro-inflammatory responses affecting neural transmission and synaptic function (20, 49). Recent research has also shown that fungi-induced changes in gut-associated lymphoid tissue further amplify immune-neuroendocrine crosstalk (50), emphasizing the complex interaction between gut mycobiota, immunity, and neuropsychiatric dysfunction in this comorbid population. Notably, a six-species fungal signature (*S. cerevisiae*, *T. asahii*, *C. albicans*, *P. ostreatus*, *Lodderomyces elongisporus*, *Wickerhamomyces anomalus*) exhibited exceptional diagnostic accuracy (AUC = 0.86), underscoring the unique utility of mycobiota profiling for identifying disease-specific microbial signatures. This performance aligns with a previous study by Xing et al., in which a decision tree-based model of five bacterial taxa achieved an AUC of 0.94 for discriminating SZ patients with and without MetS (8). Collectively, these findings highlight the diagnostic potential of gut microbiota—encompassing both bacterial and fungal communities—for SZ-MetS. Future research should prioritize validation of these mycobiota signatures in independent multicenter cohorts to facilitate their translation into clinical practice.

Our findings demonstrate significant species-specific interactions between gut mycobiota and host physiology in SZ-MetS patients, building upon and extending previous research in this field (19, 20). Most notably, we observed a strong positive correlation between *T. asahii* abundance and pro-inflammatory chemokines, particularly MIP-1α. This association aligns with established mechanisms of fungal pathogenicity, as *T. asahii* cell wall β-glucans are known to activate Dectin-1 receptors on macrophages, triggering NLRP3 inflammasome assembly and subsequent release of IL-1β and MIP-1α (33, 51). These inflammatory mediators may contribute to both the metabolic and neuropsychiatric components of SZ-MetS by promoting insulin resistance and neuroinflammation. Conversely, we found that reduced levels of *S. cerevisiae* were significantly associated with elevated IL-1β and MIP-1α. This inverse relationship supports the emerging understanding of *S. cerevisiae* as an immunomodulatory commensal, capable of attenuating TLR-mediated inflammatory responses through multiple mechanisms, including upregulation of regulatory cytokines and stabilization of gut barrier function (52). The protective effects of *S. cerevisiae* may be particularly relevant in SZ-MetS, where chronic low-grade inflammation is a hallmark feature. From a metabolic perspective, our data reveal complex fungal-host interactions. *L. elongisporus* showed a strong positive correlation with serum triglyceride levels, consistent with animal studies demonstrating its capacity to enhance hepatic lipid synthesis via PPAR-γ activation (53). Conversely, *C. albicans* abundance was inversely related to triglyceride levels, mirroring observations in NASH patients where *C. albicans* may compete for lipid nutrients (54). The immunological implications of mycobiota alterations were further underscored by our finding that *W. muriae* depletion correlated with elevated IL-8. This rare basidiomycete has been shown to promote regulatory T cell differentiation in the intestinal mucosa (55), suggesting its reduction in SZ-MetS may contribute to the disruption of immune homeostasis. Collectively, these observations support a model wherein gut mycobiota dysbiosis participates in a vicious cycle of SZ-MetS pathophysiology: fungal-driven inflammation exacerbates metabolic dysfunction, while resulting metabolic disturbances (e.g., hypertriglyceridemia) create an environment favoring further fungal dysbiosis (56). This cycle may be amplified by virulence factors such as fungal phospholipases, which directly damage host tissues and potentiate inflammatory responses (57). Our findings thus position the gut mycobiota as both a contributor to and consequence of SZ-MetS pathology, offering multiple potential intervention targets.

Building on the taxonomic and functional shifts in the gut mycobiota described above, integrating these findings with our prior characterization of gut bacterial communities in SZ-MetS patients provides a more holistic perspective on microbial dysbiosis in this comorbidity (12). Our prior work identified significant bacterial perturbations, including depletion of anti-inflammatory taxa such as *Bacteroides* and *Faecalibacterium* alongside enrichment of pro-inflammatory genera like *Escherichia-Shigella* and *Klebsiella*. These bacterial shifts are likely to interact with the fungal dysbiosis characterized in the current study, as bacterial and fungal communities in the gut maintain intricate reciprocal relationships that regulate ecosystem stability (15, 18). Mechanistically, bacterial metabolites such as SCFAs, whose production is compromised in SZ-MetS due to reduced SCFA-producing bacteria (12), play a role in constraining fungal overgrowth by modulating gut pH and reinforcing intestinal barrier integrity (30, 42). The depletion of *S. cerevisiae* observed in our current study— a species known to synergize with SCFA-producing bacteria to stabilize mucosal barriers—may thus be exacerbated by the concurrent loss of bacterial SCFA sources. Conversely, the enrichment of opportunistic fungi like *C. albicans* and *T. asahii* in SZ-MetS could disrupt bacterial homeostasis: these fungi secrete phospholipases and proteases that degrade intestinal barriers (57), creating a permissive environment for pro-inflammatory bacteria to translocate and amplify systemic inflammation. Notably, both bacterial and fungal dysbiosis in SZ-MetS correlate with elevated pro-inflammatory cytokines (e.g., IL-6, MIP-1α), suggesting a convergent impact on immune activation. For instance, bacterial lipopolysaccharides and fungal β-glucans may synergistically activate TLR4 and Dectin-1 signaling pathways (33, 51), driving the chronic low-grade inflammation that links metabolic dysfunction and neuropsychiatric symptoms. Such cross-kingdom interactions likely contribute to the “vicious cycle” of gut barrier impairment, immune dysregulation, and clinical manifestations in SZ-MetS, highlighting the need to consider multi-kingdom microbial dynamics in future mechanistic investigations. While these observations support potential bacterial-fungal crosstalk in SZ-MetS, the current study did not explicitly investigate the direct molecular or ecological mechanisms underlying such interactions. This represents an important avenue for future research, as disentangling these relationships could reveal novel therapeutic targets that simultaneously modulate both microbial kingdoms.

While our findings provide important insights, several limitations must be acknowledged. First, the cross-sectional design precludes establishing causal relationships between mycobiota alterations and disease progression. Second, while we controlled for major confounders, the potential effects of antipsychotic medications on fungal communities require dedicated investigation through medication-naïve studies. Third, our single-center design and specific ethnic composition may limit the generalizability of findings to other populations. Fourth, the diagnostic value of key functional fungal taxa identified in this study is solely based on results from the discovery cohort, with no independent validation cohort to confirm their reliability and robustness, which hinders the direct translation of these findings into clinical applications. Finally, the functional consequences of observed mycobiota changes warrant verification through mechanistic studies employing gnotobiotic models or multi-omics approaches.

## Conclusion

This study comprehensively characterizes gut mycobiota dysbiosis in Chinese SZ-MetS patients, revealing distinct taxonomic shifts including enrichment of *Candida*, *Trichosporon*, and *Issatchenkia*, alongside reduction of protective taxa like *S. cerevisiae* and *P. ostreatus*. Despite comparable global diversity, these compositional changes correlate with systemic immune dysfunction, as evidenced by elevated pro-inflammatory cytokines and species-specific associations with metabolic/clinical parameters. A six-species fungal signature demonstrates robust diagnostic accuracy, highlighting mycobiota’s potential as novel biomarkers for SZ-MetS. Collectively, these findings establish gut mycobiota dysbiosis as a critical contributor to the SZ-MetS vicious cycle. Future research should prioritize longitudinal studies to track mycobiota dynamics during disease progression, mechanistic investigations using gnotobiotic models to clarify fungal-mediated immune-metabolic crosstalk, and clinical trials evaluating *S. cerevisiae*-based probiotics to restore gut homeostasis. Additionally, integrating metatranscriptomic and metabolomic approaches to explore fungal-bacterial interactions may unveil innovative therapeutic targets for this complex comorbidity.

## Data Availability

The datasets presented in this study can be found in online repositories. The names of the repository/repositories and accession number(s) can be found below: https://www.ncbi.nlm.nih.gov/, PRJNA1280732.
